# Prescriptions

**Published:** 1896-02

**Authors:** 


### HAIR TONIC.

℞. Tinct. cantharidis....... ℥j.

Aceti distillati.......... ℥iss.

Glycerinæ................ ʒiss.

Spts. rosmarini.......... ℥iss.

Aquæ rosæ............. ad ℥viij.

M. Sig.:—To be well sponged on to the scalp night and morning.—TILBURY FOX.

### HERPES ZOSTER.

℞. Boric acid............gr. i.
Glycerine.............q. s.
Vaseline..............gr. xxx.
Cocaine hydrochlorate,
Extract of opium.aa ctgr. xxx.

M. Sig.:—Apply to the affected part.

The neuralgia following the eruption is best treated by Fowler's solution.

### IMPOTENCE.

℞. Tinct. phosphori........ ℥iss.

Tinct. canthridis....... ʒiiiss.

Elixir simplicis......ad ℥v.

M. Sig.:—One teaspoonful three or four hours before retiring. Increase the dose carefully.—VAN BUREN AND KEYES.

### INCONTINENCE OF URINE.

℞. Strychniæ sulph........ gr. j.
Pulv. cantharidis........gr. ij.
Morphiæ sulph..........gr. iss.
Ferri redacti............gr. xx.

M. Ft. pil. no. xl. Sig.:—One pill thrice daily to a child ten years old.—GROSS.

PLEURISY.

℞. Morphiæ acetatis ......gr. ss.
 Potassii acetatis......℥ss.
 Tinct. veratri viridis..♏xxiv.
 Syr. tolutani ........℥ss.
 Liq. potassii citratis..℥iiss.
M. Sig.:—A dessertspoonful every three hours. (*In dry pleurisy.*)—DA COSTA.

ACUTE RHEUMATISM.

℞. Sodii salicylatis.........℥ss.

 Tinct. lavandulæ co.... ʒiv.

 Glycerinæ ..............℥ss.

 Aquæ .... ..............ad ℥viij.

M. Sig.:—A tablespoonful every hour or two until pain and fever abate: then at longer intervals.—F. MINOT, *Mass. Gen. Hosp.*

PERICARDITIS.

℞. Hydrarg. chloridi mitis,
 Pulv. ipecac........aa gr. vj.
 Potassii nitratis... ......ʒss–j.
M. In pulv. no. xii. div. Sig.:—A powder every three hours.—HARTSHORNE.

CHRONIC CONSTIPATION.

℞. Aloes ..................gr. iv
 Strychninæ sulphat.....gr. ¼.
 Extract belladonnæ....gr. j¼.
 Ipecac pulv.............gr. vss.
M. Divide into pil. xij. Sig.:—One every evening.

BRONCHITIC ASTHMA.

℞. Potassii iodidi............ʒij.

 Ammon. carb.............ʒj.

 Tinct. lobilæ.............fʒi

 Sp. chloroformi..........fʒiv.

 Vin. ipecac .............fʒj.

 Infus. senegæ...q. s ad. f℥vj.

M. Sig.:—A tablespoonful in a wineglassful of water every four hours.

HÆMORRHOIDS.

℞. Pulv. gallæ.............gr. xx.

 Pulv. opii ...............gr. x.

 Ungt. plumbi subacetat.gr. xl.

 Ungt. simplicis.........ʒj.

M. Ft. unguentum. Sig.:—Use locally.—OESTERLEN.

